# Supplementary material for: Satisfying QTPP of Erythropoietin Biosimilar by QbD through DoE-Derived Downstream Process Engineering
Source: Pharmaceutics. 2023 Aug 4;15(8):2087. doi: 10.3390/pharmaceutics15082087 (PMC10460001; doi:10.3390/pharmaceutics15082087)
Supplement: Supplementary file 1 [file pharmaceutics-15-02087-s001.zip › pharmaceutics-2256761-SI.pdf]

pharmaceutics

MDPI

Article

Satisfying QTPP of Erythropoietin Biosimilar by QbD through DoE-Derived Downstream Process Engineering

Kakon Nag [1*], Enamul Haq Sarker [1†], Samir Kumar [1†], Sourav Chakraborty[1], Maksusdur Rahman Khan[1], Mashfiqur Rahman Chowdhury[1], Rony Roy[1], Ratan Roy[1], Bipul Kumar Biswas[1], Emrul Hasan Bappi[1], Mohammad Mohiuddin[1] and Naznin Sultana [1*]

Table S1. DoE design conditions of AFC process step along with recovery %.

Run order	Run No.	pH	[NaCl]mM	%Recovery
16	1	9.4	2000	41
13	2	5.9	2000	32
15	3	7.7	2000	60
11	4	7.6	1595	95
3	5	7	1000	32
7	6	5.4	1340	36
12	7	6.2	1645	70
2	8	7.0	1000	29
8	9	9.3	1585	55
4	10	9.4	1174	50
5	11	9.4	1174	49
10	12	7.6	1595	91
1	13	5.6	1000	32
6	14	8	1249	56
14	15	5.9	2000	50
9	16	7.6	1595	93

* ***N.B.***: Model: Quadratic, p value for model: 0.0003, p value for lack of fit: 0.0308, R^2: 0.8260, adj. R^2: 0.7390, pred. R^2: 0.4809.

Table S2. DoE design conditions of AEX process step along with recovery %.

Run order	Run	pH	[NaCl]mM	%Recovery
12	1	5	440	24
7	2	6.7	270	40
4	3	8.4	100	10
13	4	5	440	21
1	5	5	100	12
11	6	6.4	410	42
15	7	7.5	500	25
10	8	9	356	30
16	9	7.5	500	23
14	10	9	500	24
9	11	5	272	21
5	12	8.2	240	35
2	13	6.7	100	14
6	14	6.7	270	41
8	15	6.7	270	42
3	16	8.4	100	10

****N.B.***: Model: Quadratic, p value for model: <0.0001, p value for lack of fit: 0.0028, R^2: 0.9114, adj. R^2: 0.8671, pred. R^2: 0.7024.

Table S3. DoE design conditions of RPC process step along with recovery %.

Run order	Run	pH	%Acetonitrile	%Recovery
7	1	2.5	41	57
11	2	2.7	50	52
16	3	1.8	100	10
6	4	2.2	36	25
2	5	1.5	7	0
8	6	1.5	41	13
9	7	2.7	50	43
10	8	2.7	50	45
13	9	2.7	98	12
5	10	2.8	24	8
12	11	3.5	59	8
15	12	1.8	100	13
3	13	3.5	17	3
14	14	3.5	100	5
1	15	2.2	5	0
4	16	3.5	17	4

* ***N.B.***: Model: Quadratic, p value for model: 0.0009, p value for lack of fit: 0.0074, R^2: 0.7561, adj. R^2: 0.6341, pred. R^2: 0.3859.

Table S4. DoE design conditions of CEX process step along with recovery %.

Run Order	Run	pH	Buffer Volume (CV)	%Recovery
13	1	2	7	0
10	2	4.2	4.4	25
12	3	5.9	4.9	60
8	4	2	4.4	0
7	5	7	3.2	52
11	6	4.2	4.4	15
6	7	7	3.2	55
9	8	4.2	4.4	17
5	9	3.8	2.3	12
1	10	6	4	98
16	11	6.3	7	60
4	12	2	1.8	0
14	13	4.1	7	19
2	14	5.5	4	97
3	15	2	1.8	0
15	16	6.3	7	62

* ***N.B.***: Model: Cubic, p value for model: <0.0001, p value for lack of fit: 0.7473, R^2: 0.9906, adj. R^2: 0.9962, pred. R^2: 0.5791.

Table S5. Starting materials quantity, volume and concentration for 50 ml (1×) batch size.

Batch No.	Initial Quantity (mg)	Sample Volume (ml)	Initial Concentration (mg/ml)
01	53	~ 50	1.06
02	45	~ 50	0.9
03	55	~ 50	1.1
04	50	~ 50	1.0
05	54	~ 50	1.08

Table S6. AFC process adaptation data in dynamic mode for 50 ml (1×) batch size.

Batch No.	Elution Buffer pH	Elution Buffer con-Ductance (mS/cm)	Eluted Sample pH	Eluted Sample Conductance (mS/cm)	Eluted Sample Quantity (mg)	Initial Quantity (mg)	% Yield
01	7.45	123.14	7.43	122.60	42.96	53.00	81.06
02	7.24	122.21	7.21	125.25	38.56	45.00	85.69
03	7.40	125.32	7.39	124.80	44.13	55.00	80.24
04	7.56	121.61	7.52	122.64	39.68	50.00	79.36
05	7.52	124.82	7.22	123.61	42.78	54.00	79.22
Average	7.43	123.42	7.35	123.78	41.62	51.40	81.11
STDEV	± 0.12	±1.61	±0.14	±1.22	±2.38	±4.04	±2.66

Table S7. Buffer exchange process adaptation data in dynamic mode for 50 ml (1×) batch size.

Batch No.	Retentate sample pH	Retentate Sample con-Ductance (mS/cm)	Retentate Sample Quantity (mg)	Initial Quantity (mg)	% Yield
01	7.41	2.16	42.51	42.96	98.95
02	7.22	2.59	38.23	38.56	99.14
03	7.40	2.32	43.41	44.13	98.37
04	7.54	2.83	39.12	39.68	98.59
05	7.19	2.76	42.18	42.78	98.60
Average	7.35	2.53	41.09	41.62	98.73
STDEV	±0.15	±0.29	±2.27	±2.38	±0.31
Acceptance limit	7.35±0.15	2.53±0.29	-	-	98.73±0.31

Table S8. AEX process adaptation data in dynamic mode for 50 ml (1×) batch size.

Batch No.	Elution Buffer pH	Elution Buffer Conductance (mS/cm)	Eluted Sample pH	Eluted Sample Conductance (mS/cm)	Eluted Sample Quantity (mg)	Initial Quantity (mg)	% Yield
01	7.12	123.16	7.10	24.21	17.32	42.51	40.74
02	7.16	121.43	6.91	25.26	15.03	38.23	39.31
03	6.86	122.61	7.08	24.61	18.12	43.41	41.74
04	6.94	125.11	6.83	24.86	16.21	39.12	41.44
05	7.18	123.24	6.95	26.01	17.63	42.18	41.80
Average	7.05	123.11	6.97	24.99	16.86	41.09	41.01
STDEV	±0.14	±1.33	±0.11	±0.69	±1.24	±2.27	±1.03

Table S9. RPC process adaptation data in dynamic mode for 50 ml (1×) batch size.

Batch No.	Elution Buffer pH	Acetonitrile % In Elution Buffer	Eluted Sample pH	Acetonitrile % In Sample	Eluted Sample Quantity (mg)	Initial Quantity (mg)	% Yield
01	2.34	95	2.20	50	8.51	17.32	49.13
02	2.54	95	2.00	48	7.82	15.03	52.03
03	2.41	95	2.29	52	9.16	18.12	50.55
04	2.36	95	2.16	51	8.69	16.21	53.61
05	2.5	95	2.21	53	9.21	17.63	52.24
Average	2.43	95	2.17	50.80	8.68	16.86	51.51
STDEV	±0.09	0	±0.11	±1.92	±0.57	±1.24	±1.72

Table S10. CEX process adaptation data in dynamic mode for 50 ml (1×) batch size.

Batch No.	Elution Buffer pH	Elution Buffer volume (CV)	Eluted Sample pH	Eluted Sample Quantity (mg)	Initial Quantity (mg)	% Yield
01	7.22	3.5	5.71	7.65	8.51	89.86
02	6.8	3.5	5.82	6.25	7.82	79.92
03	7.15	3.5	5.68	8.47	9.16	92.45
04	6.91	3.5	5.89	6.60	8.69	75.95
05	7.26	3.5	5.93	8.39	9.21	91.11
Average	7.07	3.5	5.81	7.47	8.68	85.86
STDEV	±0.20	0	±0.11	±1.02	±0.57	±7.42

Table S11. Virus filtration process data for 50 ml (1×) batch size.

Batch No.	Filtered Sample pH	Filtered Sample Conductance (mS/cm)	Filtered Sample Quantity (mg)	Initial Quantity (mg)	% Yield
01	7.20	12.5	7.56	7.65	98.86
02	7.18	12	6.18	6.25	98.91
03	7.09	13.19	8.31	8.47	98.14
04	7.22	12.63	6.58	6.60	99.80
05	6.92	12.39	8.25	8.39	98.29
Average	7.12	12.54	7.38	7.47	98.80
STDEV	±0.12	±0.43	±0.97	±1.02	±0.65
Acceptance limit	-	-	-	-	98.80±0.65

Table S12. Sterile filtration process data for 50 ml (1×) batch size.

Batch No.	Filtered sample pH	Filtered Sample Conductance (mS/cm)	Filtered Sample Quantity (mg)	Initial Quantity (mg)	% Yield
01	7.22	12.45	7.42	7.56	98.15
02	7.18	12.11	6.16	6.18	99.69
03	7.1	13.16	8.25	8.31	99.27
04	7.21	12.59	6.50	6.58	98.84
05	6.93	12.41	8.10	8.25	98.21
Average	7.13	12.54	7.29	7.38	98.83
STDEV	±0.12	±0.39	±0.93	±0.97	±0.67
Acceptance limit	-	-	-	-	98.83±0.67

Table S13. Overall yield data of 50 ml (1×) batch size.

Batch No.	Initial Quantity (mg)	Final Quantity (mg)	% Yield
01	53.00	7.42	14.00
02	45.00	6.16	13.69
03	55.00	8.25	15.00
04	50.00	6.50	13.00
05	54.00	8.10	15.00
Average	-	-	14.14
STDEV	-	-	±0.87
Acceptance limit	-	-	14.14±0.87

Table S14. Validation of 500 ml (10×) of batch size.

Parameter	Batch 06	% Yield	Batch 07	% Yield	Batch 08	% Yield	Acceptance Limit of % yield	Observation
Starting sample volume (ml)	500	-	500	-	500	-	-	-
Starting sample concentration (mg/ml)	1.04	-	1.01	-	0.98	-	-	-
Starting sample quantity (mg)	520.00	-	505.00	-	489.00	-	-	-
Quantity of AFC elute (mg)	417.87	80.36	414.76	82.13	405.67	82.82	≥ 80%	Within limit
Quantity of TFF retentate (mg)	411.73	98.53	408.99	98.61	400.93	99.37	98.73±0.31%	Within limit
Quantity of AEX elute (mg)	164.40	39.93	167.44	40.94	169.87	39.43	≥ 40%	Within limit
Quantity of RPC elute (mg)	82.55	50.21	85.11	50.83	85.23	49.95	≥ 40%	Within limit
Quantity of CEX elute (mg)	75.01	90.87	73.36	85.19	69.36	82.20	≥ 80%	Within limit
Quantity of VF sample (mg)	73.74	98.31	72.01	98.16	68.71	98.88	98.80±0.65%	Within limit
Quantity of SF sample (mg)	72.62	98.48	71.06	98.68	67.98	99.41	98.83±0.67%	Within limit
Overall % yield		13.97		14.07		13.90	14.14±0.87%	Within limit

Table S15. Validation of 5000 ml (100×) of batch size.

Parameter	Batch 09	% Yield	Batch 10	% Yield	Batch 11	% Yield	Acceptance Limit of % Yield	Decision
Starting sample volume (ml)	5000	-	5000	-	5000	-	-	-
Starting sample concentration (mg/ml)	1.09	-	0.99	-	1.08	-	-	-
Starting sample quantity (mg)	5445	-	4950	-	5376	-	-	-
Quantity of AFC elute (mg)	4450.02	81.73	4579.17	92.51	4493.78	83.59	≥ 80%	Within limit
Quantity of TFF retentate (mg)	4406.43	99.02	4550.16	99.37	4480.27	99.70	98.73±0.31 %	Within limit
Quantity of AEX elute (mg)	1812.33	41.13	1794.21	39.43	1837.75	41.02	≥ 40%	Within limit
Quantity of RPC elute (mg)	910.318	50.23	896.2	49.95	937.28	51.00	≥ 40%	Within limit
Quantity of CEX elute (mg)	747.91	82.16	736.67	82.20	735.73	78.50	≥ 80%	Within limit
Quantity of VF sample (mg)	733.71	98.10	728.39	98.88	720.87	97.98	98.80±0.65 %	Within limit
Quantity of SF sample (mg)	724.06	98.68	724.11	99.41	715.34	99.23	98.83±0.67 %	Within limit
Overall % yield	-	13.30	-	14.62	-	13.31	14.14±0.87 %	Within limit

Table S16. Analytical findings of three different validation batches (1×, 10× and 100×).

Test parameters	Batch_1× (50 ml)			Batch_10× (500 ml)			Batch_100× (5000 ml)		
	01	02	03	01	02	03	01	02	03
General test									
Appearance	Pass	Pass	Pass	Pass	Pass	Pass	Pass	Pass	Pass
pH (7.0 ± 0.30)	6.96	6.86	6.99	6.98	6.97	6.99	6.88	6.97	6.85
Extractable volume	Pass	Pass	Pass	Pass	Pass	Pass	Pass	Pass	Pass
Uniformity of dosage units (L1≤15)	6.7	6.3	7.7	6.3	6.4	6.3	6.3	6.4	6.7
Sub-visible particles									
>10 µm	Pass	Pass	Pass	Pass	Pass	Pass	Pass	Pass	Pass
>25 µm	Pass	Pass	Pass	Pass	Pass	Pass	Pass	Pass	Pass
Identification									
Isoform pattern	Pass	Pass	Pass	Pass	Pass	Pass	Pass	Pass	Pass
Molecular mass (KDa)	~40	~40	~40	~40	~40	~40	~40	~40	~40
Peptide mapping	Pass	Pass	Pass	Pass	Pass	Pass	Pass	Pass	Pass
Glycosylation pattern	Pass	Pass	Pass	Pass	Pass	Pass	Pass	Pass	Pass
Impurity									
Aggregation	Pass	Pass	Pass	Pass	Pass	Pass	Pass	Pass	Pass
Host cell DNA (ng)	<10	<10	<10	<10	<10	<10	<10	<10	<10
Host cell protein (ng)	*bdl	*bdl	*bdl	*bdl	*bdl	*bdl	*bdl	*bdl	*bdl
Assay									
In-vitro assay	Pass	Pass	Pass	Pass	Pass	Pass	Pass	Pass	Pass
In-vivo assay (80-125%)	104	104	105	106	110	108	106	104	107
Receptor binding	Pass	Pass	Pass	Pass	Pass	Pass	Pass	Pass	Pass
Potency (80-125%)	104	107	105	106	105	107	104	103	105
Sterility	Pass	Pass	Pass	Pass	Pass	Pass	Pass	Pass	Pass
Bacterial endotoxins (EU/mL)	0.4	0.3	0.4	0.2	0.3	0.4	0.5	0.3	0.2

*bdl=Below detection limit

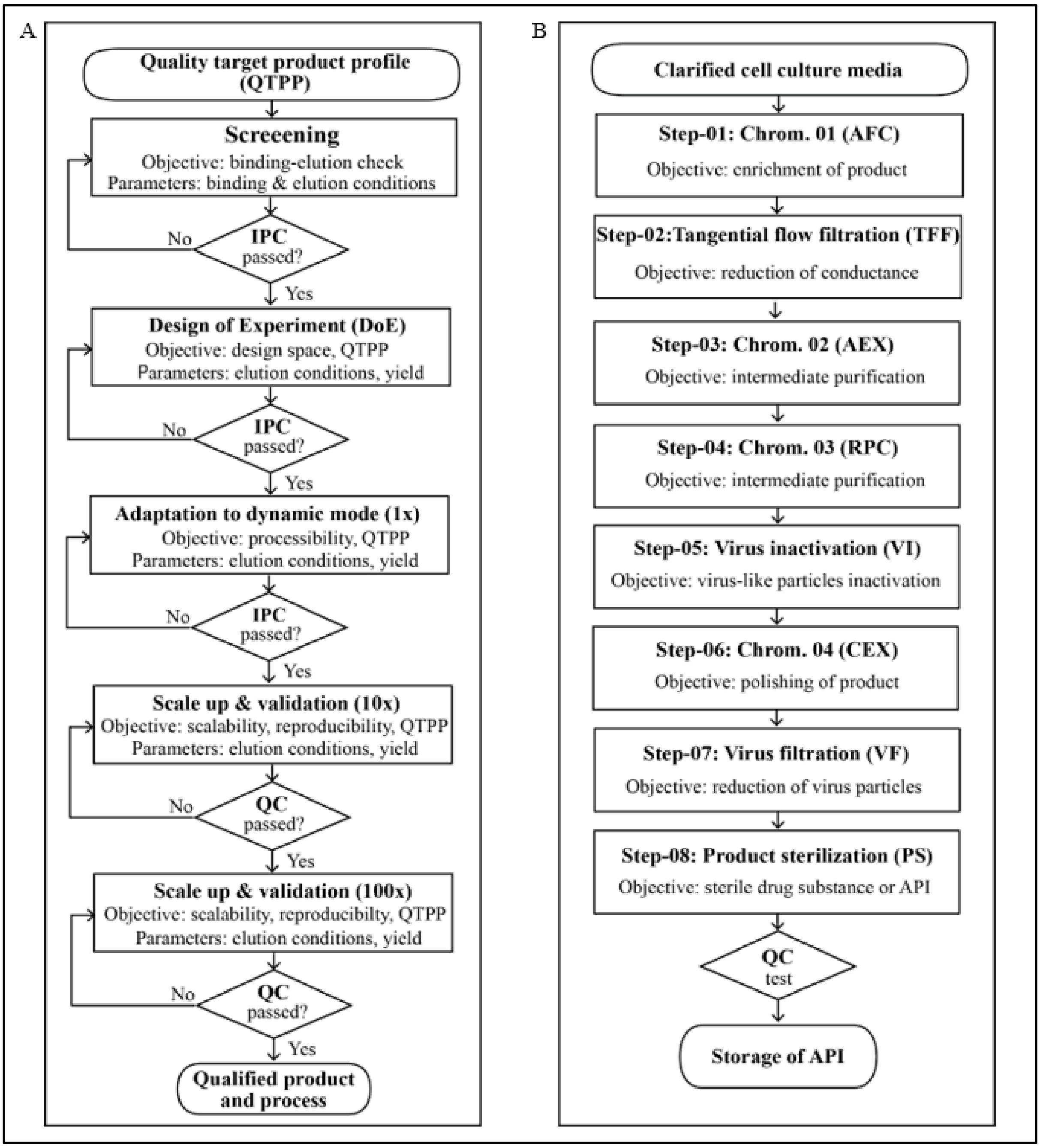

Scheme S1. Process development decision tree (A) and process flow (B) for EPO downstream processing up to ready-to-fill formulation.

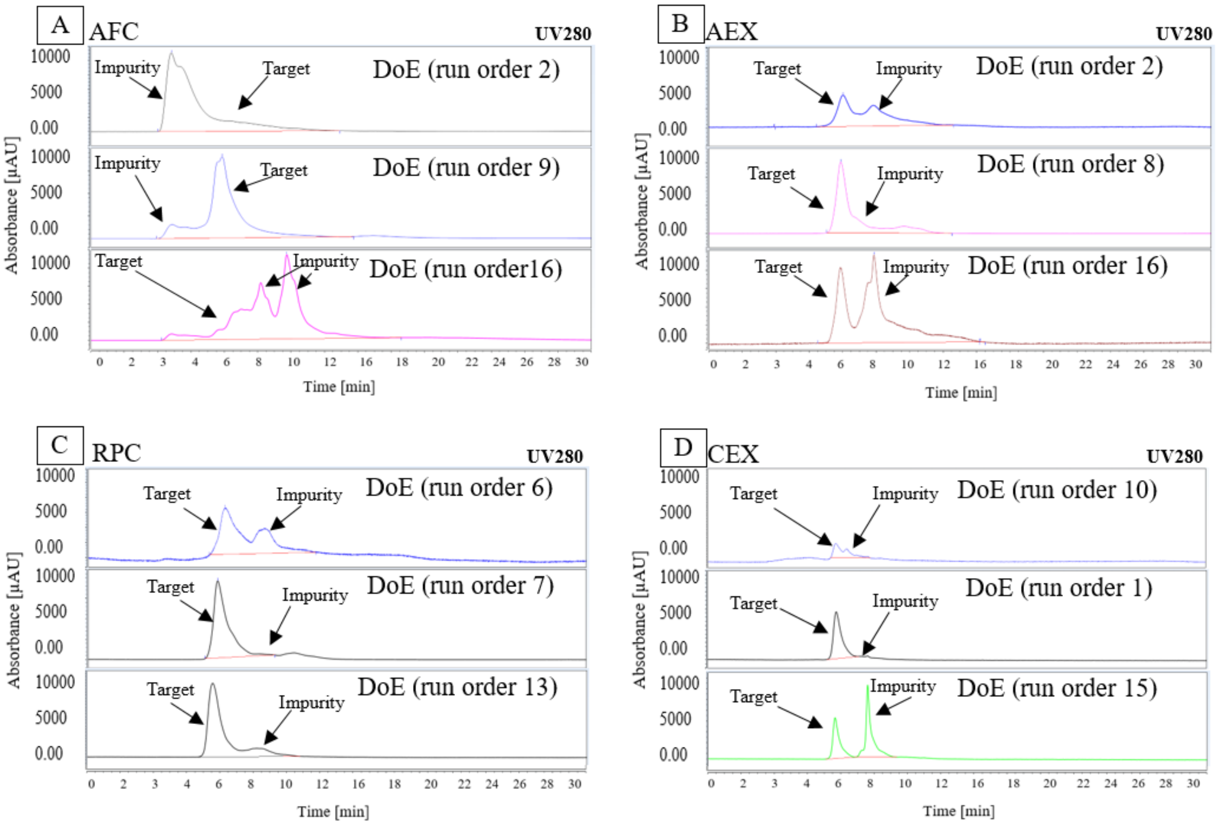

Figure S1. Size exclusion chromatography (SEC) HPLC analysis of representative samples for different process steps originated from DoE recommended runs. (A) SEC chromatograms shows target product and impurities profile of AFC samples for run order 2,9 and 16 and run order 9 shows higher recovery with least impurities than run order 2 and 16, (B) SEC chromatograms shows target product and impurities profile of AEX samples for run order 8,2 and 16 and run order 8 shows higher recovery with least impurities than run order 2 and 16, (C) SEC chromatograms shows target product and impurities profile of RPC samples for run order 6,7 and 13 and run order 7 shows higher recovery with least impurities than run order 7 and 13, (D) SEC chromatograms shows target product and impurities profile of CEX samples for run order 10,1 and 15 and run order 1 shows higher recovery with least impurities than run order 10 and 15.

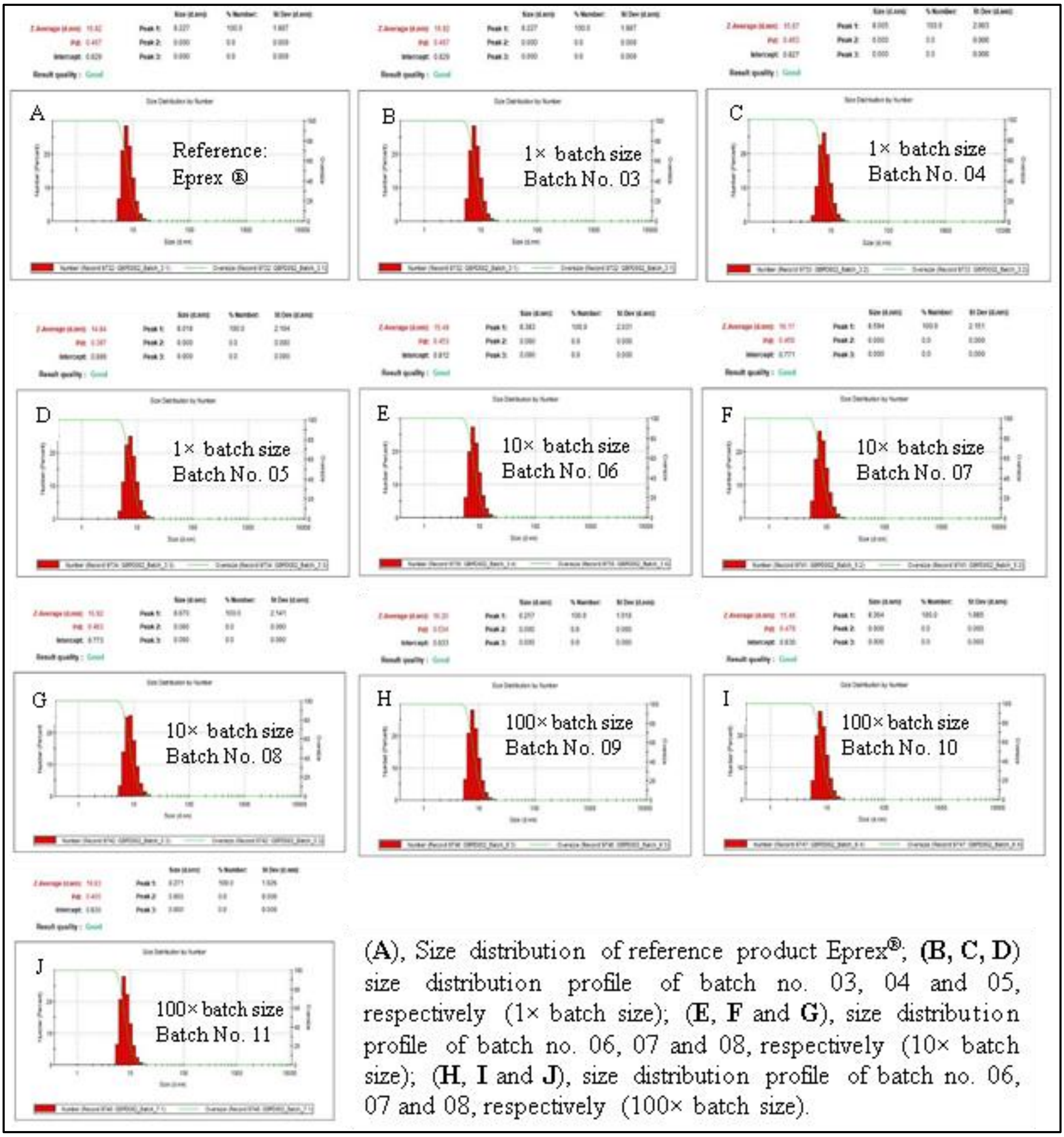

Figure S2. Comparative analysis of particle size distribution between reference product Eprex® and different batches of GBPD002.

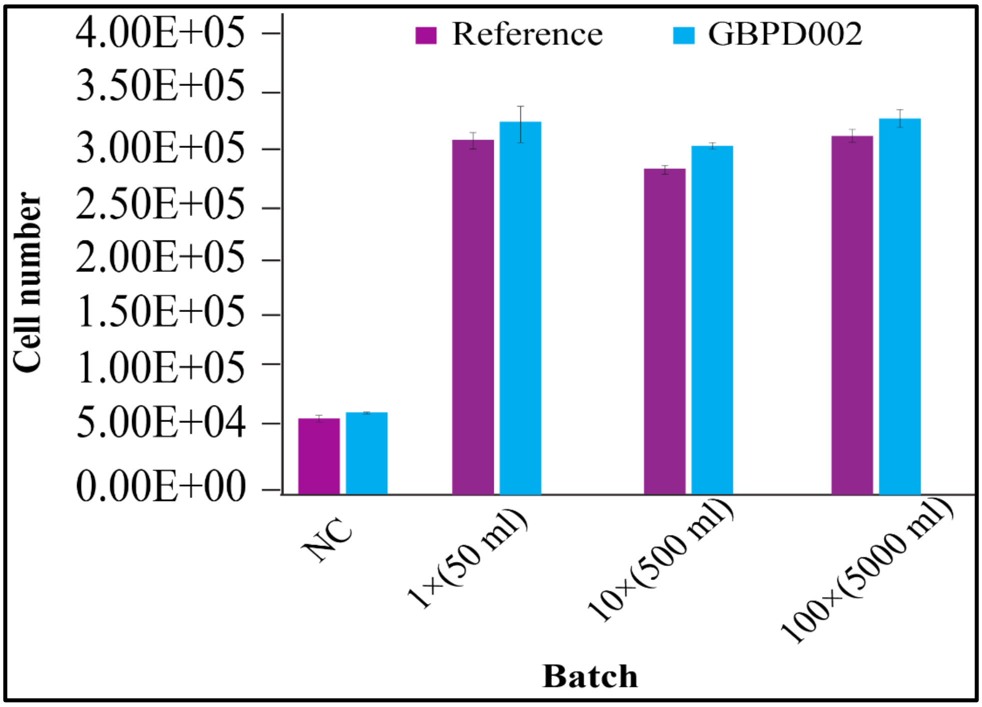

Figure S3. Comparative analysis of QTPP for Eprex® and GBPD002.Comparative bio-functionality analysis by cell culture assay.

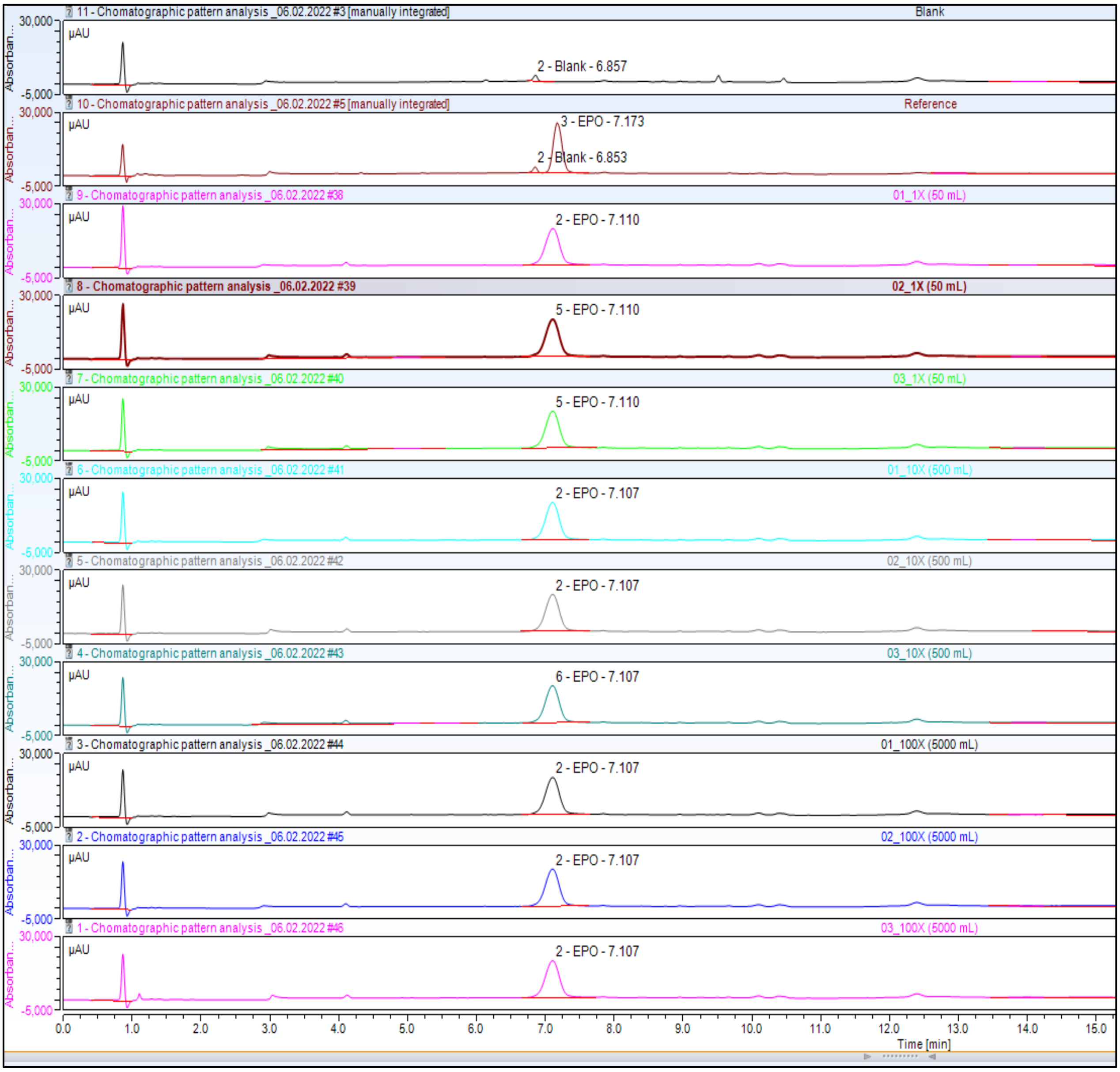

Figure S4. Determination of potency by reverse-phase chromatography and the comparative analysis of QTPP between Eprex® and GBPD002.

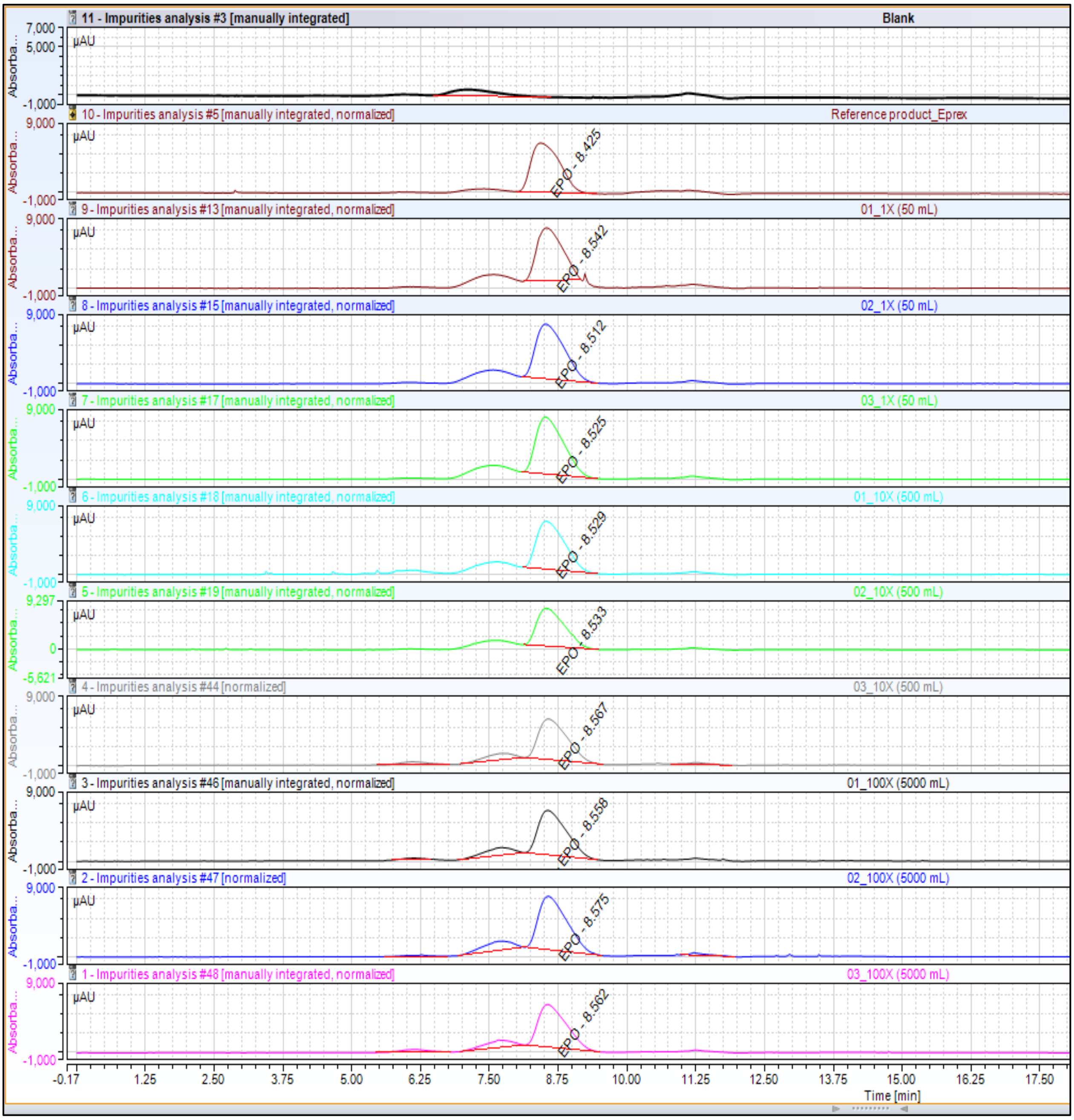

Figure S5. Impurities profile analysis by size-exclusion chromatography and the comparative analysis of QTPP between Eprex® and GBPD002.
